# Renal nerve stimulation identifies renal innervation and optimizes the strategy for renal denervation in canine

**DOI:** 10.1186/s12967-023-03919-9

**Published:** 2023-02-09

**Authors:** Hang Liu, Yidan Li, Hao Zhou, Weijie Chen, Yanping Xu, Huaan Du, Bo Zhang, Tianli Xia, Dan Li, Zhenhong Ou, Ruotian Tang, Qingsong Chen, Binyi Zhao, Yuehui Yin

**Affiliations:** 1 Department of Cardiology, The Second Affiliated Hospital of Chongqing Medical University, Chongqing, China; 2 Chongqing Cardiac Arrhythmias Therapeutic Service Center, Chongqing, China; 3 Chongqing Key Laboratory of Arrhythmia, Chongqing, China

**Keywords:** Renal denervation, Renal nerve stimulation, Renal autonomic innervation, Blood pressure

## Abstract

**Background:**

Renal denervation (RDN) was still performed without any intra-procedural method for nerve mapping. Whether renal nerve stimulation (RNS) is an efficient way to identify renal autonomic innervation and optimize the strategy for RDN remain to be worthy for further exploration.

**Methods:**

The characteristics of renal autonomic innervation at the sites with different blood pressure (BP) responses to RNS were explored. Then, dogs anatomically eligible for RDN were randomly assigned into elevated BP response ablation group, reduced BP response ablation group, and RNS-control group. The postoperative outcomes were measured at baseline and after 4 weeks follow-up.

**Results:**

The proportion of afferent sensory nerve was higher at elevated BP response sites (ERS) than reduced BP response sites (RRS) and non-response sites (NRS) (P = 0.012 and P = 0.004). Conversely, the proportion of parasympathetic nerve at RRS was the highest (RRS vs. ERS, P = 0.017; RRS vs. NRS, P = 0.023). More importantly, there was a significant correlation between systolic blood pressure changes and the area ratios of afferent sensory and parasympathetic nerve (R = 0.859; P < 0.001). In addition, ablation at BP-elevation sites can result in a significant decrease in BP and plasma norepinephrine (NE) after 4 weeks (P = 0.002; P = 0.008), while ablation at BP-reduction sites can lead to significant increases in BP and plasma NE (P = 0.016; P = 0.033).

**Conclusions:**

RNS is an effective method to identify renal autonomic innervation. It could not only help to identify optimal target sites, but also avoid ablation of sympathetic-inhibitory areas during RDN.

**Supplementary Information:**

The online version contains supplementary material available at 10.1186/s12967-023-03919-9.

## Introduction

In the last decade, catheter-based renal denervation (RDN), following the concept of regulating sympathetic activity by disrupting renal nerve fibers located in the adventitia of renal arteries, became available as a treatment option for hypertension [1]. Although RDN was controversial due to conflicting results from the SYMPLICITY HTN 1 [2], 2 [3–5] and 3 [6], the latest results of a consistent antihypertensive effect showed by SPYRAL HTN OFF/ON-MED trials [7–10] and RADIANCE HTN trials [11, 12] are encouraging and inspiring. However, some issues must still be overcome for RDN applications, such as its failure to lower blood pressure (BP) in about 20–30% of the patients in these studies [7, 9, 12]. Despite a deeper understanding of the gross anatomy and the microanatomy of renal nerves in recent years [13–15], RDN was still performed without any intra-procedural method for nerve mapping. If the therapeutic effect is interventionalist-dependent, RDN will be with a bleak outlook.

Renal nerve stimulation (RNS) may provide a meaningful method for identifying renal autonomic innervation and guiding RDN [16–18]. Our previous study has shown that the magnitude of the elevated BP responses to RNS was significantly related to the density of renal nerve fibers [17]. In other words, RNS could identify the nerve-enriched area and improve the efficacy of RDN. Recently, our team investigated and classified the phenotypes of BP response to RNS, and the results showed that renal nerve stimulation could elicit different patterns of BP response [19]. Consistent with the results of previous studies, the physiological responses of renal nerve fibers to RNS were intricate: stimulation could lead to not only an increase or no changes in BP, but also a decrease [17, 18, 20, 21]. It is generally known that renal nerves are not symmetrically distributed in the adventitia of renal arteries and surrounding tissues [13, 15]. In addition, the renal nerve bundle always simultaneously contains efferent sympathetic, afferent sensory and possible parasympathetic fibers [13, 14, 22]. Some scholars categorized these nerves as “pressor nerves” and “depressor nerves” according to the BP responses to RNS from the aspect of nervous functions [23]. Theoretically, the patterns of BP responses during RNS depend on the counter-balance between “pressor nerves” (sympathetic-excitatory) and “depressor nerves” (sympathetic-inhibitory) fibers.

However, the distribution and characteristics of these fibers at sites with elevated BP and reduced BP responses to RNS have not been fully investigated. BP changes after ablation at these sites, especially postoperative BP changes after ablation at sites with BP-reduction response, remain to be worthy for further exploration. Regrettably, it was difficult for the qualitative and quantitative analysis of the neurotypes at the ablation sites due to low/no expression of nerve markers after ablation with conventional ablation energy in our previous study [17]. Consequently, we performed ablation in a shorter time and with a lower power (induce a localized fibrotic lesion of the renal arteries, without penetrating vascular wall and damaging nerves around renal arteries) to mark the location of stimulation sites, and explored the different characteristics of renal autonomic innervation with different BP responses during RNS. Moreover, BP changes after ablation with conventional ablation energy (the renal nerves were destroyed) at sites with elevated BP and reduced BP responses to RNS were tested in the present study.

## Methods

### Experimental animal and preparation

This experiment was approved by the Animal Experimentation Ethics Committee of the Chongqing Medical University. Standard food and water feedings, as in our previous study [17], were given in Laboratory Animal Center of Chongqing Medical University throughout the experimental period. Chinese Kunming dog was chosen for the study because of its natural high blood pressure and high sympathetic tone. A series of preclinical studies involving catheter-based RDN technique have been successfully conducted on Chinese Kunming dogs by our team [17, 19, 24, 25].

Referring to the Anesthesia Guidelines issued by the University of Minnesota, dogs were fasted without water restricting overnight prior to the anesthetic event. Anesthesia was inducted by an intraperitoneal injection of 3% sodium pentobarbital (30 mg/kg, W5632, Xiya Reagent, Shandong, China). An artificial airway was established after jaw sagging and shallow breathing were observed, and then, the ventilator (WATO EX-55, Mindray, Shenzhen, China) was set to synchronized intermittent mandatory ventilation-pressure control (SIMV-PC) mode. During the procedure, sodium pentobarbital was intravenously pumped (5 mg/kg/h) to maintain the anesthesia depth, assessed by bispectral index (BIS, maintained between 50 and 70, monitored by IntelliVue MX700, Philips Medizin Systeme Boeblingen GmbH, Germany) and vital signs like pupil reflecting light, etc. Blood oxygen saturation was recorded by monitors (BeneVision N12, mindray, Shenzhen, China) and maintained no lower than 95% by continuous oxygen supply. To minimize the impact on the results, the actual anesthetic dosage was adjusted to ensure that the BIS value of each dog was maintained at a similar level during intervention and review procedure.

Bilateral femoral arteries were punctured under sterile conditions, then 2000 IU unfractionated heparin was administered followed by 200 IU every hour. Heart rate (HR), surface electrocardiogram (ECG) and invasive BP from left femoral artery were continuously monitored by a Multichannel Electrophysiology Management System (Sichuan Jinjiang Electronic Science and Technology Co., Ltd, Sichuan, China). Bilateral renal arteriography via right femoral artery was performed with a 6F JR4 Judkins catheter (Cordis Corporation, Miami, Florida, USA). Renal artery would be excluded from the study if there were any abnormalities, such as a severe distortion or stenosis.

### Experimental protocol

**Fig. 1:**
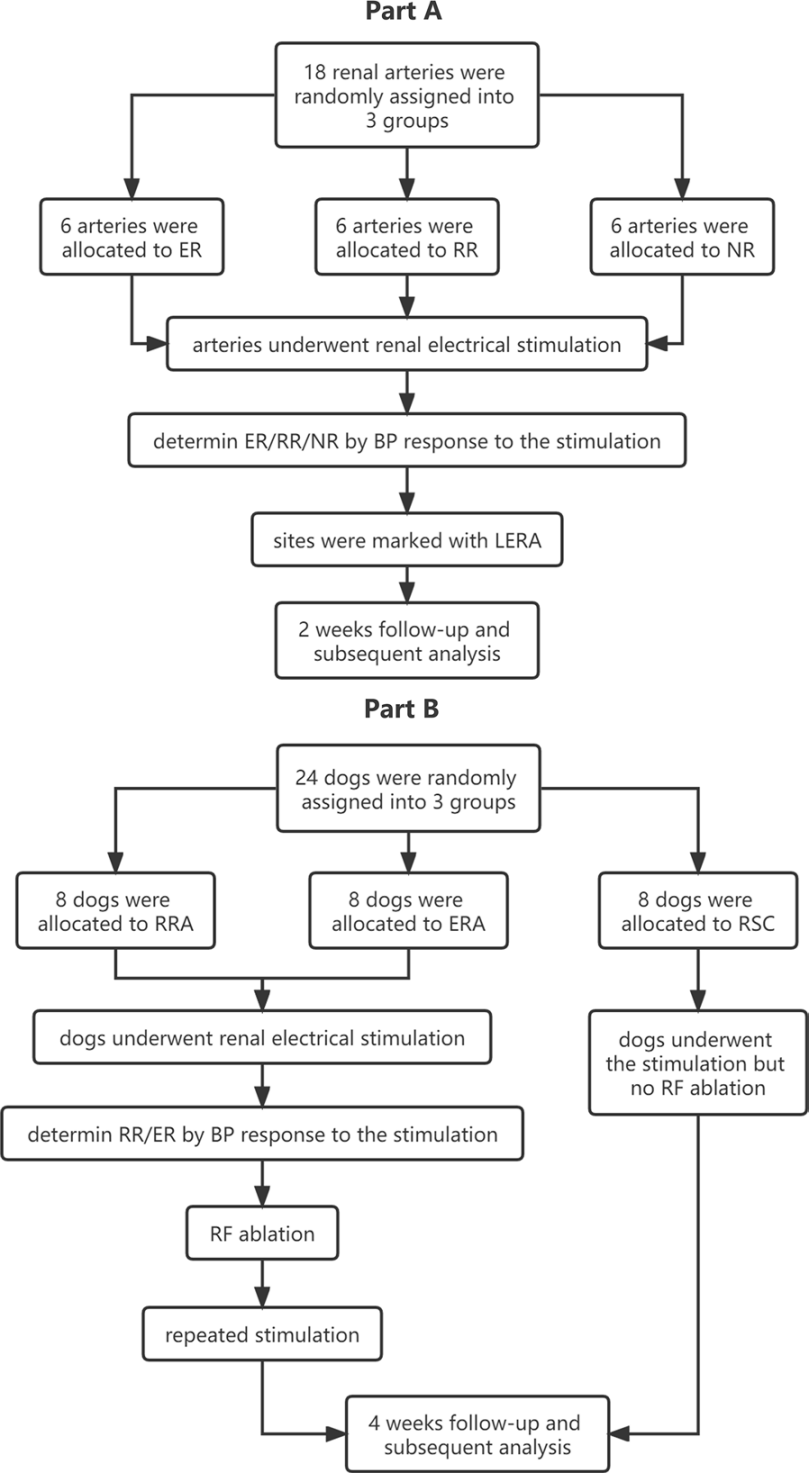
Flow-chart of the procedure. **A** the characteristics of renal autonomic innervation at these sites with different BP responses to RNS were investigated. **B** the BP changes after radiofrequency ablation at elevated BP response sites and reduced BP response sites were investigated. *ER*, elevated BP response; *RR*, reduced BP response; *NR*, non-response; *LERA*, low-energy radiofrequency ablation; *RSC*, renal stimulation control group; *ERA*, elevated BP response ablation group; *RRA*, reduced BP response ablation group; *RF*, radiofrequency

This study consisted of two parts. Part A, the characteristics of renal autonomic innervation at these sites with different BP responses to RNS were investigated. Part B, the BP changes after radiofrequency ablation at elevated BP response sites and reduced BP response sites were investigated. The flow-chart of the procedure was shown in Fig. 1.

### Part A

Available renal arteries from nine adult Chinese Kunming dogs (weighed between 20 and 30 kg and aged between 2 and 3 years) were assigned into 3 treatment groups (renal arteries, rather than dogs, were the grouping subjects of the study): elevated BP response (ER) group, reduced BP response (RR) group, and non-response (NR) group. The renal arteries in ER group underwent RNS and low-energy radiofrequency ablation (LERA, defined as inducing injury of the arterial wall without damaging nerve fibers, details are available in Additional file methods) was performed only at elevated response sites (ERS, defined as a sustained increase in the systolic blood pressure (SBP) of > 5 mmHg from baseline 20 s after the start of RNS). The renal arteries in RR group underwent RNS and LERA was performed only at reduced response sites (RRS, defined as the drop of SBP level by over 5 mmHg from baseline 20 s after the start of RNS). In NR group, RNS was delivered and LERA was performed only at non-response sites (NRS, defined as the presence of SBP slight fluctuations around baseline during RNS, fluctuation between − 5 mmHg and 5 mmHg). As described in our previous study [17, 24], we could locate the lesion generated by radiofrequency ablation in Masson’s trichrome staining due to the presence of obvious hyperplasia of collagen fibers stained blue in the ablation area. Without injury of nerve fibers during the appropriate low-energy radiofrequency ablation, the expression of nerve markers was identified for the qualitative and quantitative analysis of the neurotypes.

### Renal nerve stimulation and low-energy radiofrequency ablation

A dedicated open-irrigated ablation catheter (as our previous study described [25], AquaSense, Synaptic Medical Limited, Beijing, China) for bipolar electrical stimulation and low-energy radiofrequency ablation was introduced into the right femoral artery via a 7F sheath under fluoroscopic guidance. Electrical stimulations were performed from distal (the bifurcation) to proximal (the ostium) segments of each renal artery and delivered at the frequency of 20 Hz, energy output of 15 mA, and pulse duration of 2 ms for 60 s by using a Nerve and Muscle Stimulator (SynNuo-C4, Sichuan Jinjiang Electronic Science and Technology Co., Ltd, Sichuan, China). Eight to twelve of attempts of RNS were performed in each renal artery according to its length.

According to the pre-experimental results (details are available in Additional file results and Additional file 1: Fig. S1), we decided to choose RF energy at 3 Watts for 30 s in the proximal and 3 Watts for 20 s in the middle and distal segment as appropriate ablation parameters for LERA (the main trunk of renal artery was artificially divided into three segments of equal length under X-ray fluoroscopy). LERA was performed immediately after RNS using the same ablation catheter if the site met grouping requirements. The catheter remained in place for the duration of a cycle of RNS-LERA to ensure that the area ablated by LERA was at exactly the same site that was stimulated. Saline was irrigated at 5 ml/min to decrease the temperature of tissue-electrode interface during radiofrequency energy delivery with a Vation-Cool Pump (Sichuan Jinjiang Electronic Science and Technology Co., Ltd, Chengdu, China). Before starting the next RNS cycle, a minimum of two minutes interval between interventions were allowed to ensure that the BP return to baseline.

### Masson’s trichrome staining and immunofluorescence

Dogs were euthanized with an over-dose of sodium pentobarbital (200 mg/kg) two weeks after intervention. Bilateral renal arteries with surrounding tissues were harvested immediately and fixed in 4% phosphate-buffered paraformaldehyde for 24 h, then subjected to alcoholic dehydration, and embedded in paraffin for subsequent analysis.

Renal arteries were sectioned at approximate 5-mm intervals and each segment was cut into several 4-μm consecutive slices. One of these consecutive slices from each segment was stained with Masson’s trichrome according to the manufacturer’s instructions (G1006, Servicebio Technology, Wuhan, China) to locate the ablation area. Our previous studies have shown that hyperplasia of collagen fibers (stained blue in Masson trichrome staining) was found in ablated renal vascular wall, which could be used to locate the stimulated and ablated sites.

Tyrosine hydroxylase (TH), which is the rate-limiting enzyme in the synthesis of the dopamine and norepinephrine, was used to label efferent nerves. Calcitonin gene-related peptide (CGRP) was used for the recognition of afferent nerves. In addition, neuronal nitric oxide synthase (nNOS) and choline acetyltransferase (ChAT) were used for the recognition of parasympathetic nerves. The total area was defined as the sum of positive stained area for efferent, afferent and parasympathetic nerve markers in selected regions. The areas of specific nerve type were expressed as a percentage of the total.

Paraffin-embedded slices were deparaffinized and hydrated, then boiled intermittently in citrate buffer for 20 min for antigen retrieval. After washing and blocking, the slices were incubated with primary antibodies (tyrosine hydroxylase polyclonal antibody (TH, AB117112, Abcam, used at 1:200), monoclonal anti-calcitonin gene-related peptide (CGRP, C7113, sigma, used at 1:100), neuronal nitric oxide synthase (nNOS, AB1376, Abcam, used at 1:100), Choline acetyltransferase (ChAT,Sigma,AB143, used at 1:100) overnight at 4 °C, and then incubated with corresponding secondary antibodies (GB21403, GB21404, GB22401; Servicebio Technology, Wuhan, China) for 45 min at 37 °C. The nuclei were stained with 2-(4-amidinophenyl)-6-indolecarbamidine dihydrochloride (DAPI, G1012, Servicebio Technology, Wuhan, China) for 10 min at room temperature. Confocal laser-scanning microscope (Leica TCS STED, Leica Microsystems, Germany) was used for imaging.

### Part B

Twenty-four anatomically eligible Chinese Kunming dogs (weighed between 25 and 35 kg and aged between 3 and 4 years) were enrolled and randomly divided into 3 groups for renal denervation with conventional ablation energy: elevated BP response ablation (ERA) group, reduced BP response ablation (RRA) group, and renal stimulation control (RSC) group. The dogs in ERA group underwent RNS and ablation at elevated response sites (ERS). The dogs in RRA group underwent RNS and ablation at reduced response sites (RRS). In RSC group, dogs underwent RNS but no ablation. The definition of BP response to RNS was the same as indicated earlier in Part A.

### Renal nerve stimulation and renal denervation with conventional energy

Renal nerve stimulations were performed as described previously in Part A. Stimulation was stopped when three target sites were ablated in each renal artery. Other than that, the maximum attempts of RNS in each renal artery according to its length. These sites were ablated for 90 s with RF energy of 8–10 watts, followed by repeated stimulation. If the BP changes remained not less than 5 mmHg, an additional ablation with an added 2 watts of energy was performed for 30–40 s, followed by repeated stimulation. The catheter stayed in the same place during a cycle of stimulation-ablation-stimulation to ensure that the ablated position was exactly the stimulated site. Any new intervention was not applied until BP remained steady for at least two minutes. The impedance and temperature during ablation were monitored by the Multichannel Electrophysiology Management system. Saline was irrigated using a Vation-Cool Pump (Sichuan Jinjiang Electronic Science and Technology Co., Ltd, Chengdu, China) to ensure the temperature of the tissue-electrode interface was within 35–45 °C during RF energy delivery. The stimulation stops until it conforms to the stopping criterion (three target sites were ablated in each renal or a maximum number of attempts of RNS were performed according to its length). Penicillin was given intramuscularly after the procedure to prevent infection.

### Follow-up and measurement of plasma norepinephrine

After 4 weeks follow up, all dogs were anesthetized and underwent a surface ECG and the second intervention procedure followed by invasive femoral artery pressure monitoring, renal artery angiography. Blood samples were collected at baseline and after 4 weeks follow up. Then, dogs were euthanized and renal arteries with surrounding tissues were harvested for subsequent analysis.

Femoral vein blood samples were collected into ethylene diamine tetraacetic acid (EDTA) tubes. After precipitation at 4 °C for 2 h, the samples were subjected to high-speed centrifugation (4 °C, 3000 rpm, 15 min) for supernatant collecting. The plasma norepinephrine (NE) levels were assayed by high-performance liquid chromatography-mass spectrometry (NE standard: SN8550, Solarbio, Beijing, China).

### Masson’s trichrome staining and immunochemistry

The slice preparations were followed by previously described. One slice from each segment was stained with Masson’s trichrome to locate the ablation sites. Tyrosine hydroxylase (TH), neuronal nitric oxide synthase (nNOS) and calcitonin gene-related peptide (CGRP) were used to detect the efferent sympathetic, parasympathetic and afferent sensory nerves.

After dewaxing, gradient hydrating, antigen retrieval and endogenous peroxidase removal, the serial slices were homologous serum (AR1009, BOSTER, used at 10%; SL050, Solarbio, Beijing, China, used at 10%) blocked and then incubated overnight at 4 °C with TH polyclonal antibody (AB117112, Abcam, used at 1:500), monoclonal anti-CGRP (C7113, Sigma, used at 1:200) and nNOS (AB1376, Abcam, used at 1:200), respectively; then, they were incubated with the corresponding secondary antibodies (PV-6000, PV-9000, Origene; A0181, Beyotime) at 37 °C for 30 min. DAB solution (G1212, Servicebio Technology, Wuhan, China) and hematoxylin dye (G1004, Servicebio Technology, Wuhan, China) were used successively for chromogenic reaction and staining. A fluorescence microscope (OLYMPUS, BX53, Japan) was used for imaging. Absent or low expression indicated that the nerves were completely or partially destroyed.

### Statistics

Continuous variables were expressed as mean ± standard deviation (SD) or median with range when appropriate. Categorical variables were reported by frequencies and percentages. The differences of variables among 3 groups were analyzed with the use of one-way ANOVA, followed by post hoc analysis with LSD-t test to evaluate the differences between individual variables. If homogeneity of variances was violated, the differences of variables among 3 groups were analyzed using Welch’s ANOVA, followed by post hoc analysis with Games-Howell test. Differences of SBP for each 10-s phases were analyzed by two-way repeated measures ANOVA. Post hoc pairwise comparisons were applied when a significant interaction effect was observed. Comparisons of SBP changes before and during RNS were performed using the paired t test. Two-sided p < 0.05 was defined as statistical significance. Pearson’s correlation was used to assess the correlation. All statistical analyses were performed with SPSS statistical software (version 23.0, IBM Corps., Armonk, NY).

## Results

### Part A

#### Autonomic responses to renal nerve stimulation

After angiography, a total of 16 renal arteries were included in the study. The total number of marked sites underwent LERA was 68, including 15 in the ERS (from 3 renal arteries), 18 in the RRS (from 5 renal arteries), and 35 (from 8 renal arteries) in the NRS. The maximum SBP level, at ERS, was increased from 173.5 ± 14.8 to 194.9 ± 19.0 mmHg (P < 0.001). On the contrary, SBP at RRS decreased from 182.1 ± 7.2 to 155.2 ± 12.5 mmHg (P < 0.001). At NRS, SBP changed from 175.9 ± 22.2 to 179.8 ± 23.4 mmHg (P < 0.001), greater SBP-elevation was observed at ERS than at NRS (21.4 ± 8.7 vs 3.8 ± 4.1 mm Hg, P < 0.001). There was a significant decrease in SBP at RRS compared with that at ERS and NRS (− 26.8 ± 12.3 vs 21.4 ± 8.7 mmHg and − 26.8 ± 12.3 vs 3.8 ± 4.1 mmHg, both P < 0.001).

#### Renal autonomic innervation under different blood pressure response patterns

ChAT was used as a marker of the parasympathetic nervous system. In addition to ChAT, nNOS was used as another marker of parasympathetic innervation. As shown in Additional file 1: Fig. S2, immunofluorescent staining demonstrated that nNOS staining largely coincided with the ChAT staining in the renal nerves. For this reason, nNOS was selected as a parasympathetic marker for subsequent analysis in this study.

**Fig. 2:**
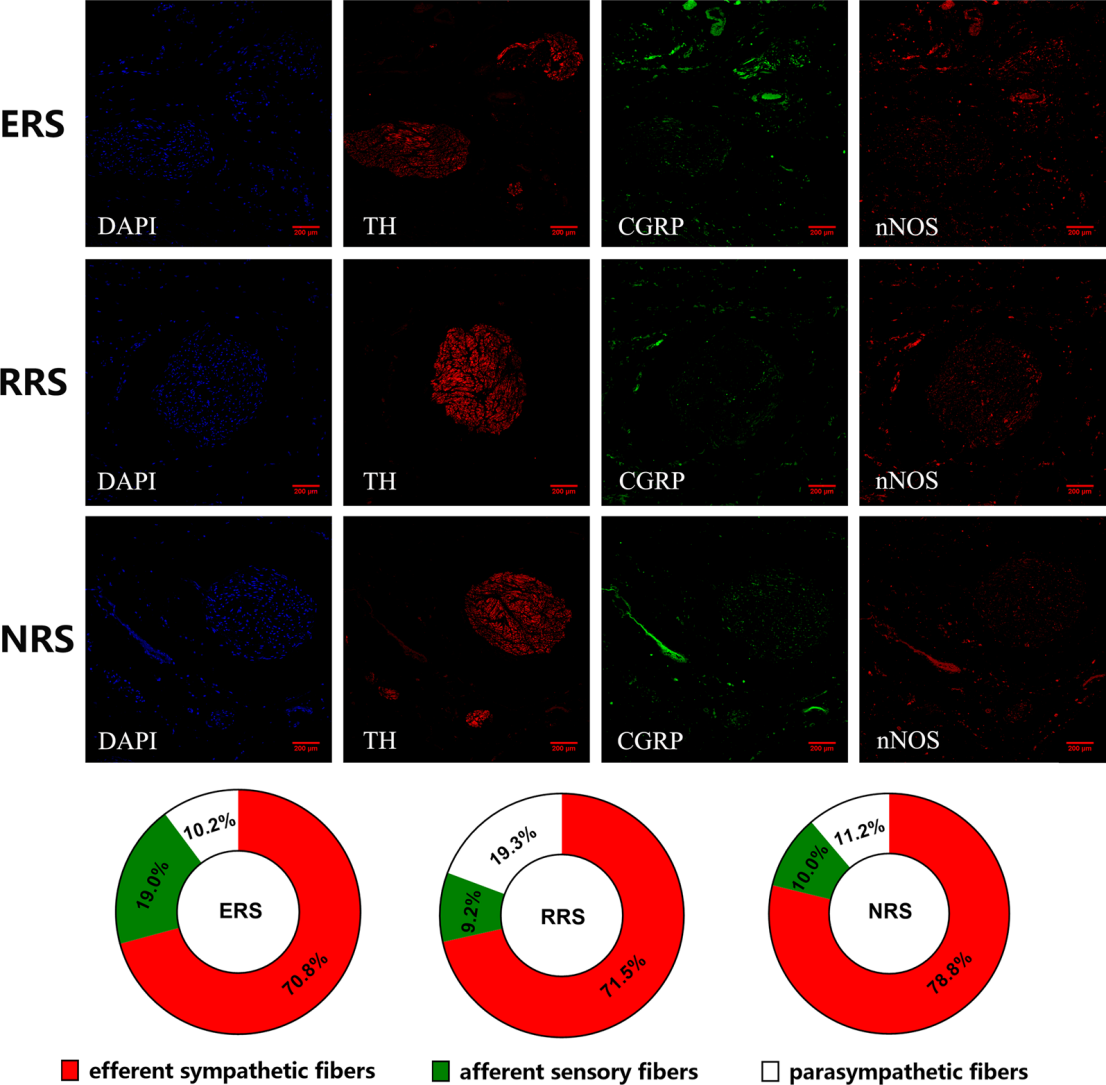
The proportion of efferent sympathetic, afferent sensory and parasympathetic nerve fibers among ERS, RRS and NRS. Renal efferent sympathetic nerves (red, labeled by TH), renal afferent sensory nerves (green, labeled by CGRP) and parasympathetic nerve fibers (red, labeled by nNOS) in one nerve bundle, and cell nuclei (blue, labeled by DAPI) were identified

As shown in Fig. 2, for ERS, proportions of efferent sympathetic nerve, afferent sensory nerve, and parasympathetic nerve were 70.8% (± 6.4%), 19.0% (± 4.7%) and 10.2% (± 1.9%), respectively. At RRS, the efferent sympathetic nerve was 71.5% (± 9.6%), the afferent sensory nerve was 9.2% (± 2.7%) and the parasympathetic nerve was 19.3% (± 6.9%). The corresponding proportions at NRS were 78.8% (± 10.7%), 10.0% (± 5.4%) and 11.2% (± 5.3%), respectively.

No significant statistical difference was found in the proportions of efferent sympathetic nerve among ERS, RRS and NRS (P = 0.251). The proportion of afferent sensory nerve was higher at ERS than at RRS and NRS (P = 0.012 and P = 0.004). There was no difference in the proportion of afferent sensory nerve between RRS and NRS (P = 0.798). The proportion of parasympathetic nerve at RRS was the highest (RRS vs. ERS, P = 0.017; RRS vs. NRS, P = 0.023), and no significant statistical difference was found between ERS and NRS (P = 0.723).

### Correlation between the BP response to RNS and renal autonomic innervation

**Fig. 3:**
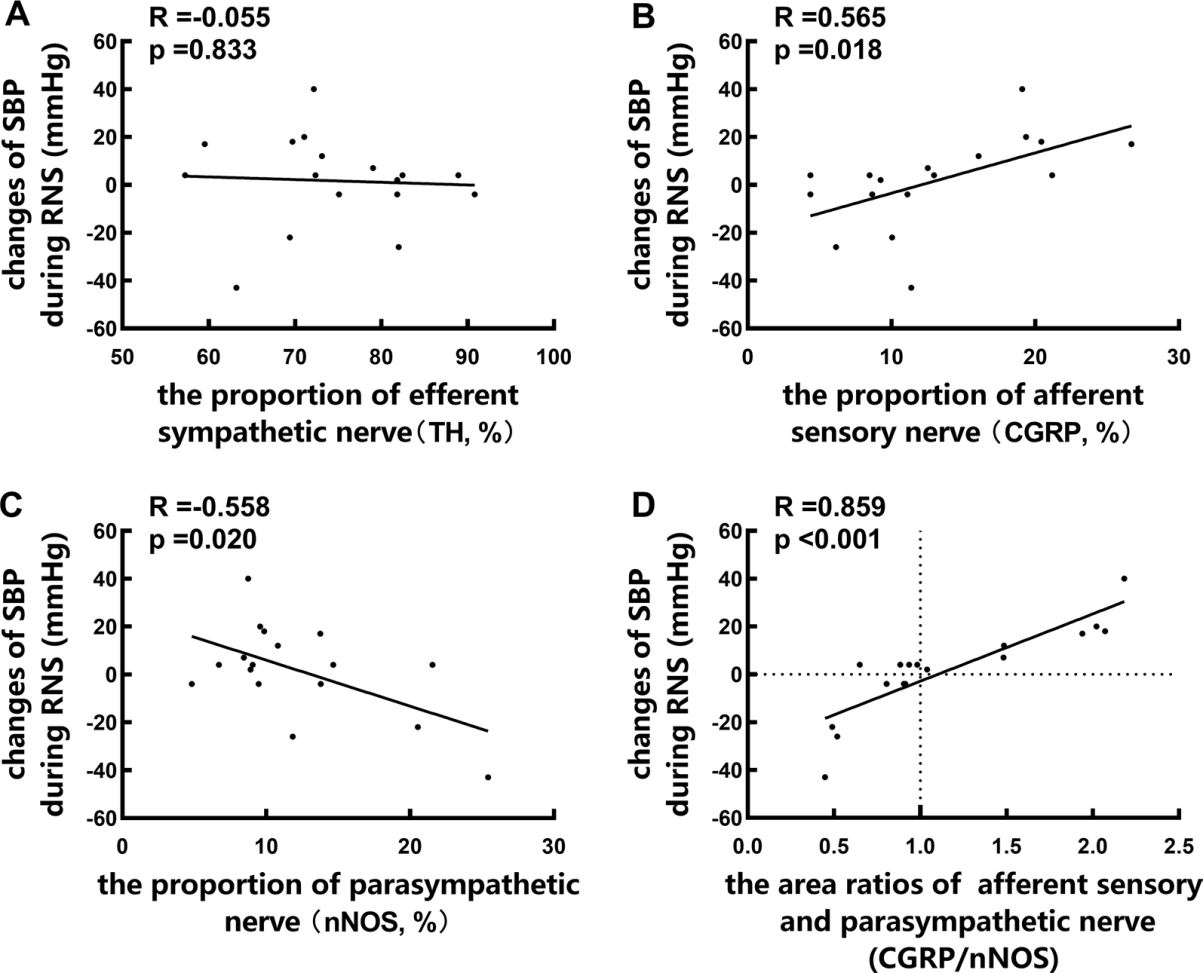
The correlation Between the BP response to RNS and renal autonomic innervation. **A** indicated no correlation between SBP changes and proportions of efferent sympathetic nerve; **B** showed the maximum SBP changes during RNS were correlated with proportions of afferent sensory nerve; **C** showed the maximum SBP changes during RNS were correlated with proportions of parasympathetic nerve and **D** showed a significant correlation between SBP changes and the area ratios of afferent sensory and parasympathetic nerve

As shown in Fig. 3, the maximum SBP changes during RNS were correlated with proportions of afferent sensory nerve and parasympathetic nerve (R = 0.565; P = 0.018 and R = 0.558; P = 0.020). It is noteworthy that, there was a significant correlation between SBP changes and the area ratios of afferent sensory and parasympathetic nerve (R = 0.859; P < 0.001), but no correlation between SBP changes and proportions of efferent sympathetic nerve (R = − 0.055; P = 0.833).

### Part B

#### Autonomic responses of sites to renal stimulation

The stimulation elicited a BP-change response at 269 sites. There were 127 RRS, the distributions of which were 38.6%, 37.0% and 24.4% at the proximal, middle and distal of the main trunk of the renal artery, respectively. While, there were 142 ERS: 31.7%, 42.9%, 25.4% were at the proximal, middle and distal segments. No significant differences showed at baseline (30 s before each intervention) among groups in SBP (172.3 ± 20.9 vs. 171.5 ± 23.3 vs. 178.0 ± 26.1 for ERA vs. RRA vs. RSC, P = 0.835) and HR (189.4 ± 18.7 vs. 174.6 ± 42.8 vs. 175.1 ± 15.0 for ERA vs. RRA vs. RSC, P = 0.287).

**Fig. 4:**
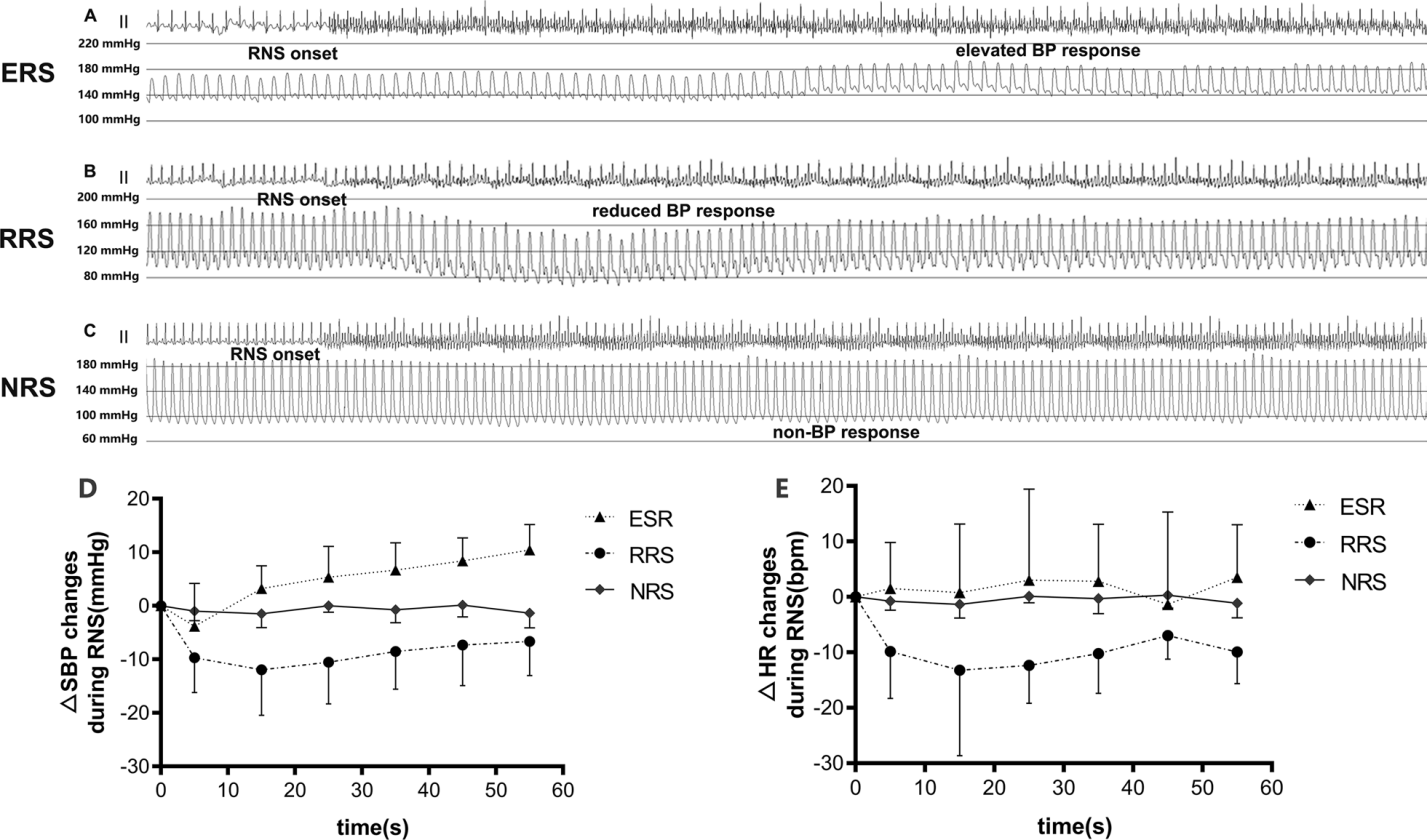
Representative images of BP response to RNS and trajectories of heart rate and BP changes. **A** showed the representative image of elevated BP response site, **B** showed the representative image of reduced BP response site, **C** showed the representative image of non-response site, **D** showed the SBP response to RNS during 60-s period, **E** showed the HR response to RNS during 60-s period

For analysis, each RNS of 60-s period was subdivided into 10-s phases. Results from 2-way repeated measures ANOVA indicated a notable difference among the three different patterns of BP response (P < 0.001). RNS increased the mean SBP of ERA by − 3.8 ± 8.0, 3.2 ± 4.2, 5.3 ± 5.8, 6.7 ± 5.1, 8.4 ± 4.3 and 10.4 ± 4.7 mmHg (P = 0.089, P = 0.122, P = 0.014, P = 0.002, P < 0.001, P < 0.001; respectively) from baseline to the first, second, third, fourth, fifth, and sixth 10 s during RNS. Meanwhile, the mean SBP of RRA decreased by − 9.7 ± 6.5, − 11.9 ± 8.5, − 10.5 ± 7.8, − 8.5 ± 7.0, − 7.3 ± 7.6 and − 6.6 ± 6.4 mmHg (P < 0.001, P < 0.001, P < 0.001, P < 0.001, P = 0.001, P = 0.001; respectively) at each time period during RNS. The mean SBP at RSC showed no significant difference from the baseline to all time periods (changed by − 1.0 ± 1.8, − 1.5 ± 2.6, 0 ± 1.2, − 0.8 ± 2.4, − 0.1 ± 2.2 and − 1.4 ± 2.7 mmHg (P = 0.645, P = 0.463, P = 1, P = 0.687, P = 0.946; P = 0.432 respectively). Trajectories of systolic blood pressure changes compared to baseline were shown in Fig. 4.

Regarding the maximum SBP changes to the stimulation before ablation among targeted sites undergoing ablation, the SBP at RRA decreased from 174.2 ± 19.7 to 151.3 ± 16.0 (P < 0.001). In contrast, the SBP at ERA increased from 171.7 ± 23.8 to 183.8 ± 25.2 (P < 0.001). However, the SBP at RSC had no apparent changes (178.0 ± 26.1 vs 175.9 ± 26.8, P = 0.105). The maximum SBP changes among the 3 patterns of BP responses were significantly different (P < 0.001).

The heart rate in RRA tended to decrease during RNS. However, taken as a whole, results from 2-way repeated measures ANOVA indicated no statistically significant difference was observed (including the time effect, overall intervention effect, and time-intervention interaction effect; P > 0.05 for all). Trajectories of heart rate changes compared to baseline were shown in Fig. 4. Changes of heart rate data for each group and each time point were presented in Additional file 1: Table S1.

#### Renal nerves destruction following ablation

Compared with the non-ablated sites, these three neural markers all showed no/lower expression at ablated sites, indicating that the renal nerves were effectively destroyed. Representative picture and quantitative analysis were in Additional file 1: Fig. S3.

During ablation, the impedance in the RRA group decreased from 203.5 ± 29.0 Ω to 171.9 ± 30.2 Ω (P < 0.001) and that in the ERA group decreased from 207.9 ± 20.6 Ω to 187.7 ± 24.1 Ω (P < 0.001). There was no obvious difference in the impedance decrease between the ERA and RRA groups (P = 0.133).

#### Postoperative blood pressure changes

**Fig. 5:**
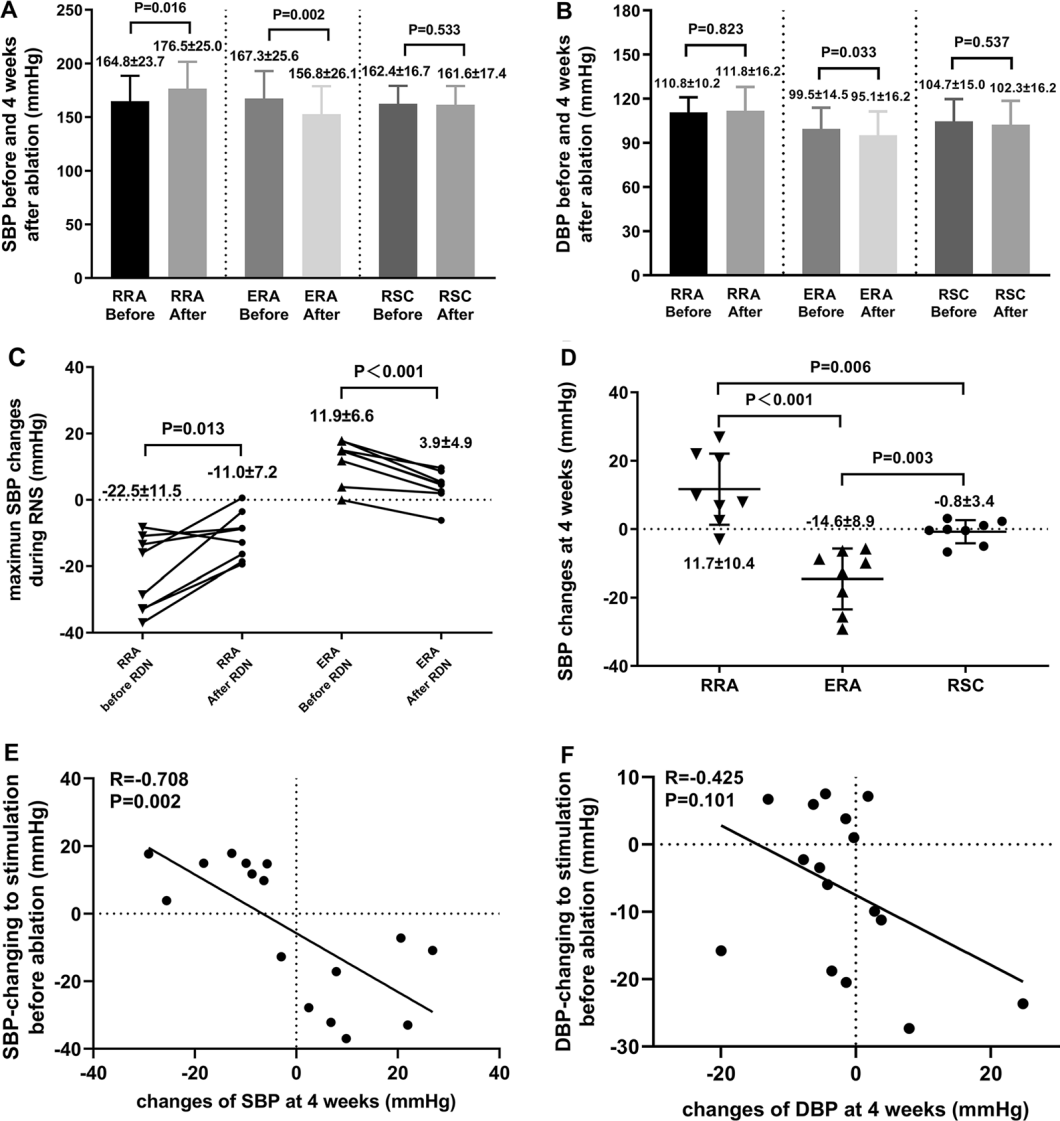
Blood pressure (BP) changes and correlation analysis. **A** levels of SBP before and 4-week after ablation among the 3 groups. **B** levels of diastolic blood pressure (DBP) before and 4-week after ablation among the 3 groups. **C** maximum SBP changes to the stimulation before and immediately after ablation in the RRA and ERA groups. **D** SBP changes at 4-week follow-up among the 3 groups. **E** correlation between SBP changes at 4-week follow-up and SBP changes to the stimulation before ablation. **F** correlation between DBP changes at 4-week follow-up and DBP changes to the stimulation before ablation

There were no significant differences in SBP and diastolic blood pressure (DBP) (P = 0.902 for SBP, P = 0.234 for DBP) among three groups at baseline (before RNS and RDN were performed). No significant differences in the number of ablated sites between the RRA (4.4 ± 1.0) and ERA (3.9 ± 1.1) groups (P = 0.376). Four weeks after the intervention, the SBP was dramatically increased in the RRA group, while significantly decreased in the ERA group (Fig. 5A, D). Nevertheless, the SBP in the RSC group after 4 weeks was similar to that at baseline (Fig. 5A, D). Furthermore, BP changes during RNS were correlated the difference between post- and preoperative level of SBP (Fig. 5E). No obvious correlation was observed between the DBP changes after 4 weeks and the DBP-change response to the stimulation before ablation (Fig. 5F).

#### The changes of plasma norepinephrine level

**Fig. 6:**
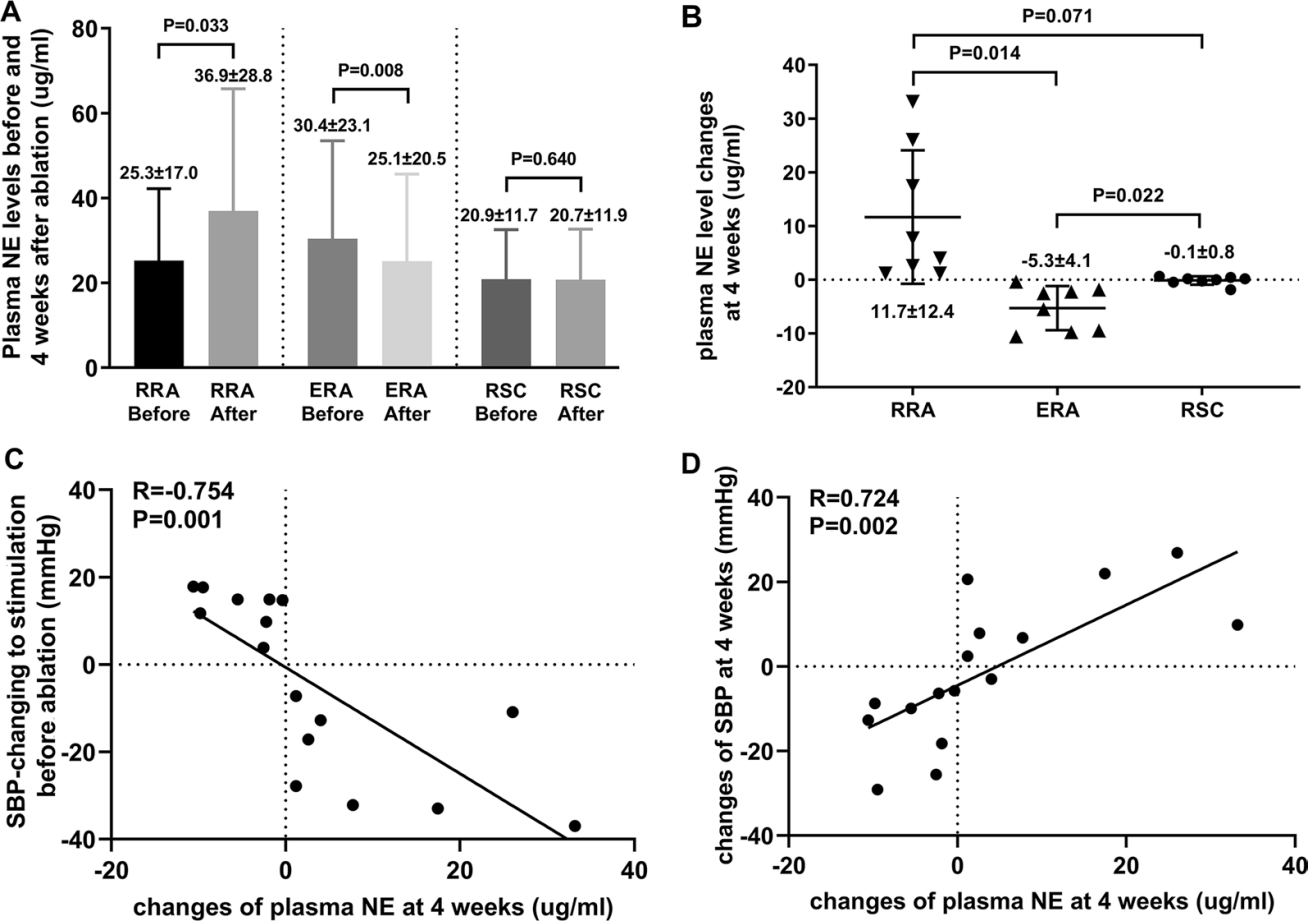
Plasma norepinephrine (NE) changes and correlation analysis. **A** plasma NE levels before and 4 weeks after ablation. **B** plasma NE changes at 4 weeks after ablation. **C** correlation between plasma NE changes at 4 weeks and systolic blood pressure (SBP) changes to the stimulation before ablation. **D** correlation between plasma NE and SBP changes at 4 weeks

The plasma NE concentration at baseline did not significantly differ among three groups (P = 0.578). The plasma NE level in the RRA group showed a dramatic elevation after 4-week follow-up. In contrast, the ERA group showed marked reduction in the plasma NE level at 4 weeks. Meanwhile, the plasma NE level in the RSC group after 4 weeks was similar to that at baseline (Fig. 6A, B).

Moreover, the maximum systolic BP changes during RNS were correlated with the difference between post- and preoperative level of plasma NE. The plasma NE changes were significantly correlated with the SBP changes after 4 weeks (Fig. 6C, D).

## Discussion

In this study, we performed ablation in a shorter time and with a lower power (induce a localized fibrotic lesion of the renal arteries, without penetrating vascular wall and damaging nerves around renal arteries) to mark the location of stimulation sites, and explored the different characteristics of renal autonomic innervation with different BP responses during RNS. More importantly, the effects of ablating these sites with opposite BP responses to RNS were investigated for the first time. The main findings were as follows: (1) The proportion of afferent sensory nerve was higher at ERS than RRS and NRS. Conversely, the proportion of parasympathetic nerve at RRS was the highest. (2) The maximum SBP changes during RNS were correlated with proportions of afferent sensory nerve and parasympathetic nerve. More importantly, there was a significant correlation between SBP changes and the area ratios of afferent sensory and parasympathetic nerve. (3) Ablation at BP-reduction sites showed a dramatic elevation in postoperative BP and plasma NE.

RDN was still performed without any intra-procedural method for nerve mapping, which was also one of the main reasons that RDN failed to lower blood pressure (BP) in about 20%-30% of the patients. Therefore, technological and procedural factors need to be further explored to improve outcome of RDN. RNS may provide a meaningful method for identifying the optimal target sites during RDN. It is important to clarify the correlation between BP responses to RNS and renal autonomic innervation. Indeed, we had tried to identify the relationship but failed [17]. It was difficult for the qualitative and quantitative analysis of the neurotypes at the ablation sites due to the low/no expression of nerve markers after ablation with RF energy of 10 Watts for 90 s in our previous study [17]. In order to solve this problem, we created a more suitable ablation parameter to mark the locations of stimulation sites. We found that low-energy radiofrequency ablation at 3 Watts for 20 s and 3 Watts for 30 s were the appropriate ablation parameters, at which partial arterial wall injury could be induced without damaging the nerve fibers (more details were showed in Additional file methods and results).

The BP responses to RNS in this study were consistent with some previous studies [17, 20, 21, 25–29] that the physiological responses of renal nerve fibers to RNS were implicated i.e. stimulation could lead to not only an increase and no changes in BP, but also a decrease. With low-energy radiofrequency ablation for marking, we further assessed the correlation between BP responses to RNS and renal autonomic innervations. It turned out that efferent sympathetic nerve is dominant in all the renal nerve bundles and no significant statistical difference was found in the proportions of efferent sympathetic nerve among ERS, RRS and NRS. The proportion of parasympathetic nerve at RRS was the highest, while the proportion of afferent sensory nerve was higher at ERS than RRS and NRS.

Although the underlying pathophysiological mechanisms of BP response to RNS have not been fully clarified yet, our results provide additional information on possible reasons for those differences. It now seems very likely that the efferent sympathetic nerve would not be responsible for the instantaneous regulation of BP response to RNS. Firstly, this immediate and substantial BP response always occurred within 10 to 20 s during RNS which is comparable with effects induced by an enhanced sympathetic nervous activity through stellate ganglion stimulation in canine studies as reported previously [30]. Stimulation of renal efferent nerves potentially regulated arterial pressure secondary to the increased tubular sodium reabsorption, renin secretion and systemic RAAS-activity [31, 32]. Theoretically, activation of renal efferent nerve would take more time to induce the BP changes. Secondly, although efferent nerves have the highest proportion, no statistical difference was found among ERS, RRS and NRS. If that was the case, then, afferent sensory and parasympathetic fibers were most likely to affect the BP response to RNS. The maximum SBP changes during RNS were correlated with proportions of afferent sensory nerve and parasympathetic nerve. More importantly, there was a significant correlation between SBP changes and the area ratios of afferent sensory and parasympathetic nerves. These results provide the basis for the hypothesis that the patterns of BP responses during RNS depend on the counter-balance between “pressor nerves” (sympathetic-excitatory fibers) and “depressor nerves” (sympathetic-inhibitory fibers).

We already know that renal efferent sympathetic activity participates in renin release, sodium retention and reduced renal blood flow, which contribute to the development of hypertension [31, 33, 34]. Ablation of renal afferent nerves can decrease arterial pressure by attenuating central sympathetic outflow to peripheral circulation system [35]. Previous studies have confirmed that injury of efferent sympathetic and afferent nerves may be beneficial to the treatment of hypertension [36–38]. However, the functional role of renal sympathetic-inhibitory fibers is poorly known. Disruption of these nerves may be detrimental for the anti-hypertensive effect of RDN.

We speculated that predominant ablation at sympathetic-excitatory sites (exert a BP rise during RNS) which pressor nerves are predominant might have greater potential for efficacy and optimization of RDN. Sites do not exert a BP rise (or even a BP fall) may reflect areas of convergence of sympathetic-inhibitory (depressor) nerves, the ablation of which should ideally be avoided. The results of the present study for the first time provide experimental evidence to this speculation. Consistent with previous studies, ablation at sites with BP-elevation responses to the stimulation significantly reduced the postoperative BP and plasma NE levels in this study. In contrast, ablation at site with BP-reduction response to RNS could lead to a significant increase in postoperative BP as well as plasma NE level. That is why we emphasize identifying renal autonomic innervation and utilizing RNS as a guide for selecting the appropriate ablated sites.

Heart rate was measured as an indicator of autonomic activity. In this study, the heart rate in RRA tended to decrease during RNS. However, taken as a whole, no statistically significant difference was observed (including the time effect, overall intervention effect, and time-intervention interaction effect). This is in agreement with previously published data [19, 39] including our own studies [19]. Esler et al. [40] demonstrated that HR in hypertension correlated directly with cardiac noradrenaline spillover, but not with renal noradrenaline spillover or adrenaline secretion. Therefore, the variance in HR was mainly attributable to differences in cardiac sympathetic activity. Previous studies have proposed that acute HR oscillations during RNS were most likely derived from a combination of afferent renal sympathetic nerve signaling enhancing the central sympathetic tone and baroreflex, vagally mediated response to changes in BP. Of note, a limitation of our study is that HR has only been assessed 1 min during RNS. It is likely that a longer intervention and recording period is required to observe HR changes.

In the light of current progress of RDN, the sustained drop in BP was slight and the number of non-responders or patients with elevated BP was large, which remained to be clarified [7–9, 11, 12, 41]. Although there are many improvements and developments in RDN techniques, such as increased number of electrodes (unipolar catheter changed to multipolar catheter or spiral catheter) and improvement in ablation position (combined main renal artery plus branch [42–44], there is currently no way to locate the renal nerve anatomically during RDN, or to distinguish the nerve types functionally. Renal nerve stimulation, as we have shown, could identify renal autonomic innervation both anatomically and functionally and optimizes the strategy during renal denervation.

## Limitations

Firstly, there are numerous arguments for a parasympathetic innervation of the kidney. Regrettably, it still lacks commonly recognized and accepted markers of renal parasympathetic nerves. In this study, we used two commonly used neural markers to label parasympathetic fibers, but both nNOS and ChAT signals partially coincided with the CGRP. Hence, it was difficult to equate the nNOS and ChAT signal positive nerves to parasympathetic fibers. It might be more appropriate to name these nerves (induced a sustained BP drop during RNS) sympathetic-inhibitory fibers or depressor nerves according to its physiological functions. Therefore, these questions whether the kidney receives innervation from parasympathetic nervous system and whether the depressor nerve is in fact the parasympathetic nerve warrant further investigation. Secondly, for technical reasons, some sites marked by LERA have not been included in the statistical analysis. Reasons for this included the absence of renal nerves in some slices and the failure of acquisition of some sites marked by LERA during the histological section. Overall, the sample sizes in the included trials were small and further studies with larger sample sizes are required to validate the results of the present study.

## Conclusions

In conclusion, RNS is an effective method to identify renal autonomic innervation. It could not only help to identify optimal target sites, but also avoid ablation of sympathetic-inhibitory areas during RDN.

## Supplementary Information

**Additional file 1: Figure S1.** Representative pictures of Masson’s trichrome stained sections. The red arrows indicate production of collagen. **Figure S2.** Representative immunofluorescence image for renal parasympathetic nerves (green, labeled by ChAT；red, labeled by nNOS) in one nerve bundle, cell nuclei (blue, labeled by DAPI). **Figure S3.** Representative image for renal nerve after RDN. A, Representative Masson’s staining image for ablated sites. The red arrow indicates collagen hyperplasia stained blue in ablated area. B, C and D, representative immunohistochemical staining image for the corresponding slices. B, neuronal nitric oxide synthase (nNOS) staining (labeling parasympathetic nerves); C, calcitonin gene-related peptide (CGRP) staining (labeling sensory afferent nerves); D, tyrosine hydroxylase (TH) staining (labeling efferent sympathetic nerve); E, quantitative analysis for nerve markers. **Table S1.** Changes of heart rate for each group and each time point

## Data Availability

The datasets used and/or analyzed during the current study are available from the corresponding author on reasonable request.

## Abbreviations

RNS: Renal nerve stimulation
RDN: Renal denervation
BIS: Bispectral index
ER: Elevated BP response
RR: Reduced BP response
NR: Non-response
LERA: Low-energy radiofrequency ablation
TH: Tyrosine hydroxylase
CGRP: Calcitonin gene-related peptide
nNOS: Neuronal nitric oxide synthase
ChAT: Choline acetyltransferase
RF: Radiofrequency
SBP: Systolic blood pressure
DBP: Diastolic blood pressure
