# Supplementary material for: Renal nerve stimulation identifies renal innervation and optimizes the strategy for renal denervation in canine
Source: J Transl Med. 2023 Feb 9;21:100. doi: 10.1186/s12967-023-03919-9 (PMC9912587; doi:10.1186/s12967-023-03919-9)
Supplement: Supplementary file 1 — Additional file 1: Figure S1. Representative pictures of Masson’s trichrome stained sections. The red arrows indicate production of collagen. Figure S2. Representative immunofluorescence image for renal parasympathetic nerves (green, labeled by ChAT；red, labeled by nNOS) in one nerve bundle, cell nuclei (blue, labeled by DAPI). Figure S3. Representative image for renal nerve after RDN. A, Representative Masson’s staining image for ablated sites. The red arrow indicates collagen hyperplasia stained blue in ablated area. B, C and D, representative immunohistochemical staining image for the corresponding slices. B, neuronal nitric oxide synthase (nNOS) staining (labeling parasympathetic nerves); C, calcitonin gene-related peptide (CGRP) staining (labeling sensory afferent nerves); D, tyrosine hydroxylase (TH) staining (labeling efferent sympathetic nerve); E, quantitative analysis for nerve markers. Table S1. Changes of heart rate for each group and each time point [file 12967_2023_3919_MOESM1_ESM.docx]

**Additional files**

**Renal Nerve Stimulation Identifies Renal Autonomic Innervation and Optimizes the Strategy for Renal Denervation in Canine**

Hang Liu ^a,b,^*; Yidan Li ^a,b,^*; Weijie Chen ^a,b^; Hao Zhou ^a,b^; Yanping Xu ^a,b^; Huaan Du ^a,b^; Bo Zhang ^a,b^; Tianli Xia ^a,b^; Dan Li ^a,b^; Zhenhong Ou ^a,b^; Ruotian Tang ^a,b^; Qingsong Chen ^a,b^; Binyi Zhao ^a,b^; Yuehui Yin ^a,b,c^

From the ^a^Department of Cardiology, the Second Affiliated Hospital of Chongqing Medical University, Chongqing, China; ^b^Chongqing Cardiac Arrhythmias Therapeutic Service Center, Chongqing, China; ^c^Chongqing Key Laboratory of Arrhythmia, Chongqing, China.

*** Drs. Liu and Li contributed equally to this work and are joint first authors.**

**Address for correspondence:**

Prof. Yuehui Yin, MD. FESC,

Department of Cardiology, the Second Affiliated Hospital of Chongqing Medical University, Chongqing Cardiac Arrhythmias Therapeutic Service Center, Chongqing, China. Chongqing Key Laboratory of Arrhythmia, Chongqing, China.

No. 288 Tianwen Avenue, Nan’an District, Chongqing, 401336, China.

Tel：+8613508335502; Fax：+86 023-63693766;

E-mail: yinyh@hospital.cqmu.edu.cn; yinyh63@163.com

**Brief title:** RNS Identifies Renal Innervation and Optimizes RDN.

# Additional file methods

**Investigation of suitable ablation parameter to mark the locations of stimulation sites**

**Study Protocol：**

In 6 renal arteries from the first 3 dogs, catheter-based RDN was performed from distal (the bifurcation of renal artery) to proximal (the ostium of renal artery) segments, using a 6F open-irrigated ablation catheter. Eight to ten lesions were created in each renal artery according to its length. The radiofrequency (RF) power of ablation at each renal artery were delivered at 2 watts for 10 seconds, 2 watts for 20 seconds, 2 watts for 30 seconds, 3 watts for 20 seconds, 3 watts for 30 seconds and 4 watts for 30 seconds to determine the appropriate ablation parameters for low-energy radiofrequency ablation (LERA, defined as inducing injury of the arterial wall without damaging nerve fibers) to mark the locations of stimulation sites. We could locate the lesion generated by radiofrequency ablation in Masson’s trichrome staining due to the presence of obvious hyperplasia of collagen fibers stained blue in the ablation area. Without injury of nerve fibers during the appropriate low-energy radiofrequency ablation, the expression of nerve markers was identified to illuminate the proportion of efferent sympathetic, afferent sensory and parasympathetic nerve fibers.

**Tissue acquisition：**

Dogs were euthanized with an over-dose of sodium pentobarbital (200 mg/kg) two weeks after operation. Bilateral renal arteries with surrounding tissues were harvested immediately and fixed in 4% phosphate-buffered paraformaldehyde for 24 hours, then subjected to alcoholic dehydration, and embedded in paraffin for subsequent analysis.

# Additional file results

As shown in Supplemental Figures 1.A, RF energy at 1 Watts for 20 seconds, 2 Watts for 10 seconds, 2 Watts for 20 seconds, 2 Watts for 30 seconds all produced no vascular-associated fibrosis.

As shown in Supplemental Figures 1.B, RF energy at both 3 Watts for 20s and 3 Watts for 30s successfully induced fibrosis of the renal arteries, without penetrating vascular wall and damaging nerves. However, RF energy at 3 Watts for 30s may penetrated the vascular wall at a few sites. To balance the success of marking and the induction of nerve injury, we decided to choose RF energy at 3 Watts for 30s in the proximal and 3 Watts for 20s in the middle and distal segment as appropriate ablation parameters for low-energy radiofrequency ablation (LERA).

# Additional file Figures


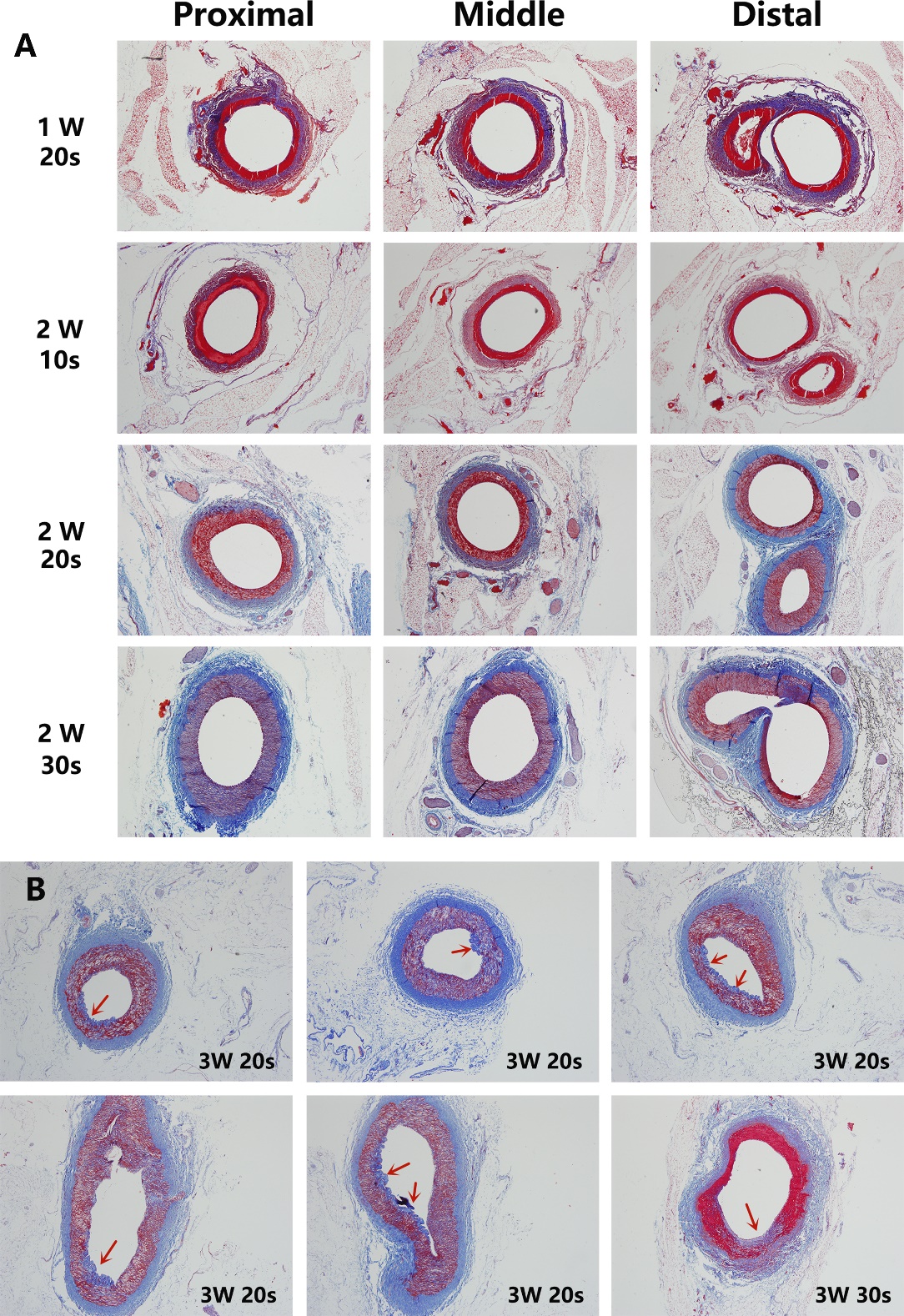


**Figure S1. Representative pictures of Masson’s trichrome stained sections. The red arrows indicate production of collagen.**


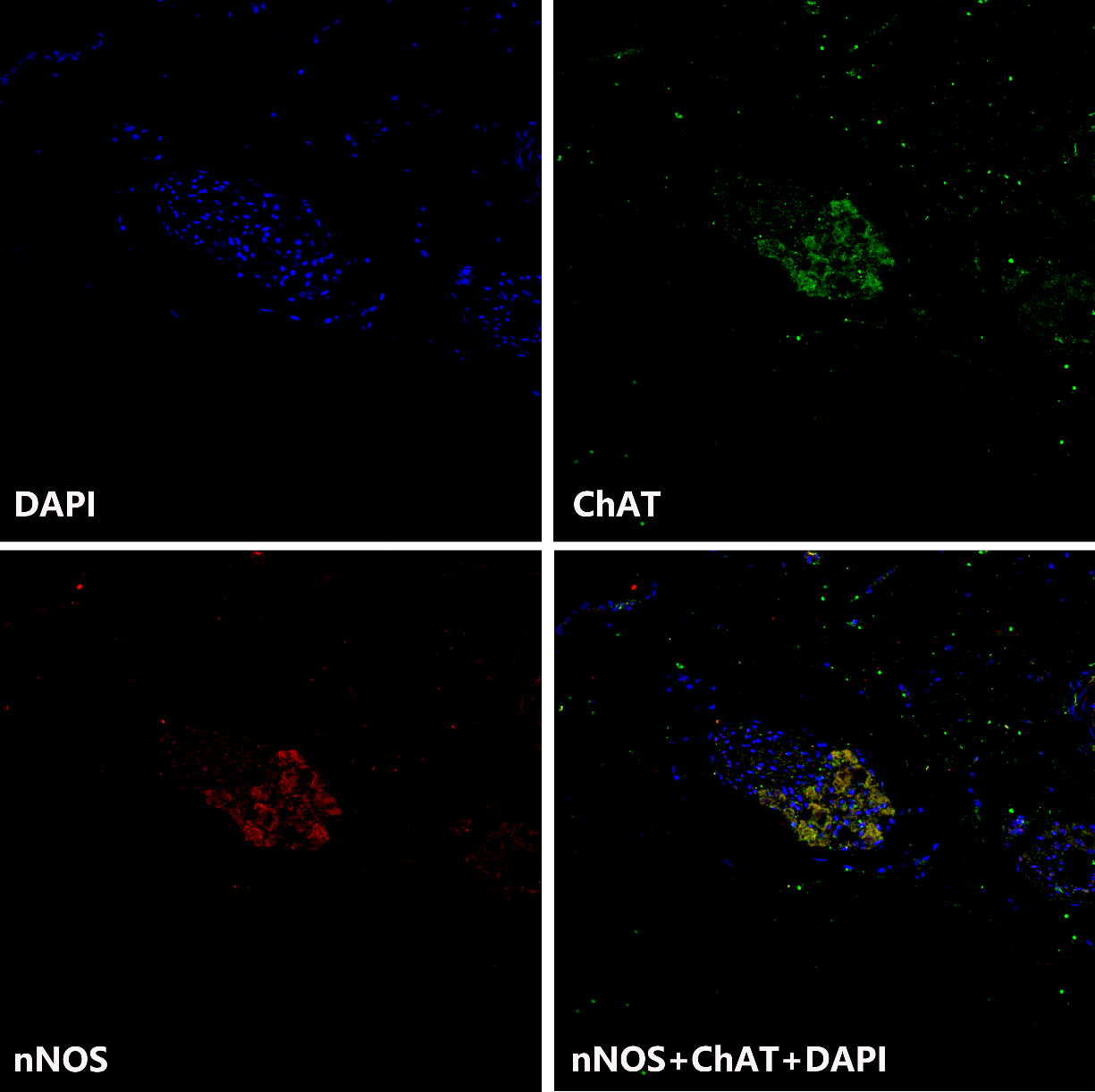


**Figure S2.** Representative immunofluorescence image for renal parasympathetic nerves (green, labeled by ChAT；red, labeled by nNOS) in one nerve bundle, cell nuclei (blue, labeled by DAPI).


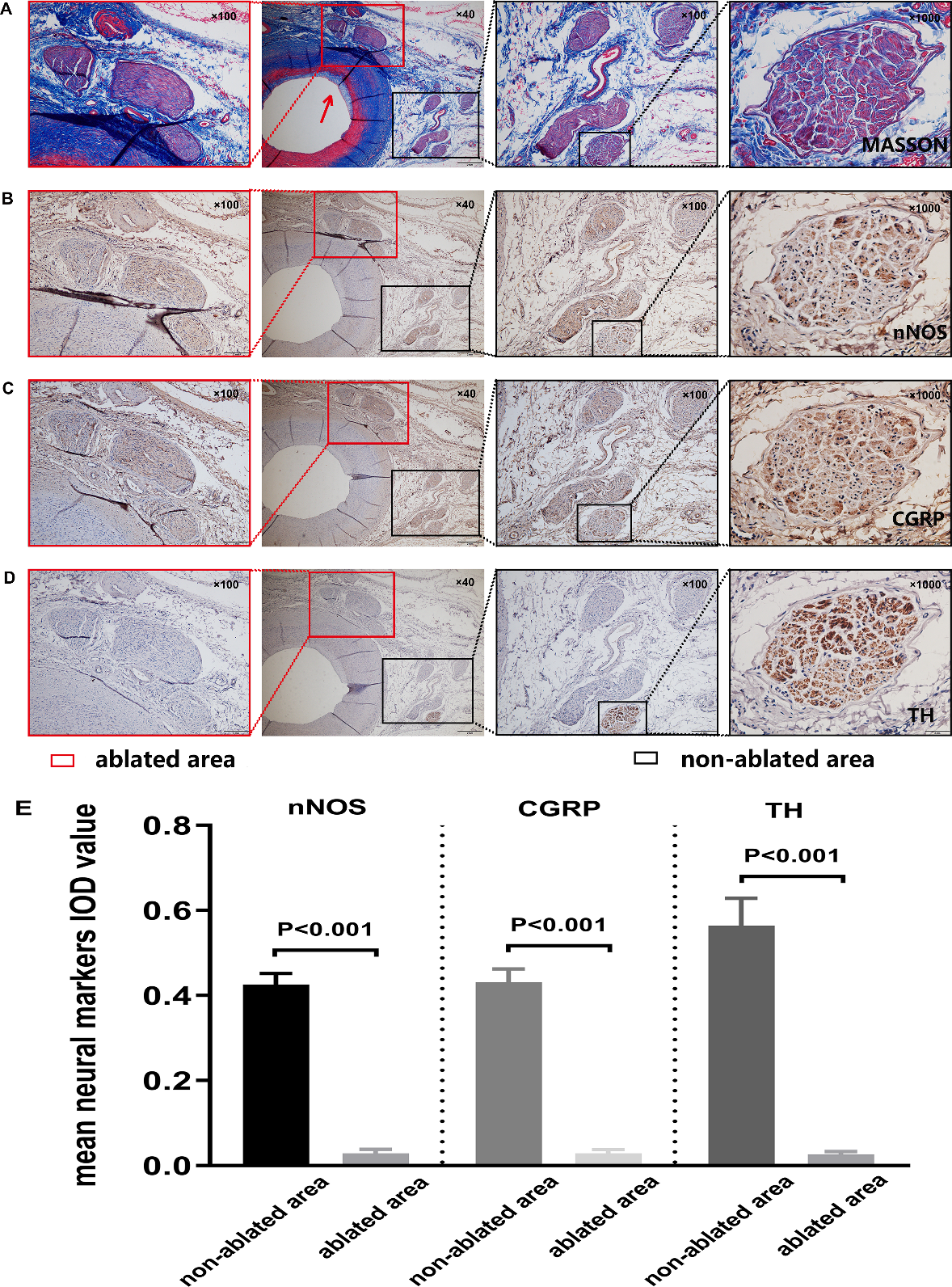


**Figure S3.** Representative image for renal nerve after RDN. A, Representative Masson’s staining image for ablated sites. The red arrow indicates collagen hyperplasia stained blue in ablated area. B, C and D, representative immunohistochemical staining image for the corresponding slices. B, neuronal nitric oxide synthase (nNOS) staining (labeling parasympathetic nerves); C, calcitonin gene-related peptide (CGRP) staining (labeling sensory afferent nerves); D, tyrosine hydroxylase (TH) staining (labeling efferent sympathetic nerve); E, quantitative analysis for nerve markers.

# Additional file tables

**Table S1. Changes of heart rate for each group and each time point**

| Group | ∆ HR （bpm） | | | | | |
| --- | --- | --- | --- | --- | --- | --- |
|  | 0s-10s | 10s-20s | 20s-30s | 30s-40s | 40s-50s | 50s-60s |
| RRA | -9.9±8.5 | -13.3±15.4 | -12.3±6.8 | -10.2±7.2 | -7.0±4.3 | -9.9±5.7 |
| ERA | 1.5±8.3 | 0.8±12.4 | 3.0±16.4 | 2.8±10.3 | -1.4±16.7 | 3.5±9.5 |
| RSC | -0.8±1.6 | -1.4±2.5 | -0.1±1.2 | -0.3±2.7 | -0.3±2.3 | -1.2±2.6 |

Values are present as mean ± SD. ∆ HR =change in heart rate from baseline;
